# Mermithid nematodes found in adult *Anopheles* from southeastern Senegal

**DOI:** 10.1186/1756-3305-5-131

**Published:** 2012-06-28

**Authors:** Kevin C Kobylinski, Massamba Sylla, William Black, Brian D Foy

**Affiliations:** 1 Arthropod-borne Infectious Disease Laboratory, Department of Microbiology, Immunology and Pathology, Colorado State University, 1692 Campus Delivery, Fort Collins, CO 80523-1692, USA

**Keywords:** Mermithidae, *Anopheles*, Senegal

## Abstract

**Background:**

Over two dozen mermithid nematodes have been described parasitizing mosquitoes worldwide, however, only two species were found in Africa. Mermithid nematodes kill their mosquito host upon emergence, which suggests that they could be developed as biological control agents of mosquitoes. Both *Romanomermis culicivorax* and *Romanomermis iyengari* have been reared for mass release to control numerous *Anopheles* species vector populations, and in one instance this may have led to reduced malaria prevalence in a human population.

**Methods:**

*Anopheles* mosquitoes were collected during a malaria study in southeastern Senegal. Two different adult blood fed mosquitoes had a single mermithid nematode emerge from their anus while they were being held post-capture. Primers from the 18 S rDNA were developed to sequence nematode DNA and screen mosquitoes for mermithid DNA. 18 S rDNA from the Senegalese mermithid and other mermithid entries in GenBank were used to create a Maximum Parsimony tree of the Mermithidae family.

**Results:**

The mermithid was present in 1.8% (10/551) of the sampled adult *Anopheles* species in our study area. The mermithid was found in *An. gambiae* s.s., *An. funestus*, and *An. rufipes* from the villages of Ndebou, Boundoucondi, and Damboucoye. Maximum parsimony analysis confirmed that the nematode parasites found in *Anopheles* were indeed mermithid parasites, and of the mermithid sequences available in GenBank, they are most closely related to *Strelkovimermis spiculatus*.

**Conclusions:**

To our knowledge, this is the first report of mermithids from adult *Anopheles* mosquitoes in Senegal. The mermithid appears to infect *Anopheles* mosquitoes that develop in diverse larval habitats. Although maximum parsimony analysis determined the mermithid was closely related to *Strelkovimermis spiculatus*, several characteristics of the mermithid were more similar to the *Empidomermis* genus. Future mermithid isolations will hopefully allow: formal taxonomic identification, laboratory colonization, determination of life history traits and species specificity, and characterize its usefulness as a biological control agent.

## Background

Nematodes from the family Mermithidae (Enoplea: Mermithida) parasitize terrestrial and aquatic invertebrates, including many insect disease vector species [1]. Many insect disease vectors are parasitized by mermithid nematodes during their aquatic larval stage, including blackflies and mosquitoes [2]. Seven genera of Mermithidae are parasites of mosquitoes: *Culicimermis, Empidomermis, Hydromermis, Octomyomermis, Perutilimermis, Romanomermis*, and *Strelkovimermis*[3], and over 25 species have been described [4].

While numerous mermithids have been found in mosquitoes on every continent except Antarctica [4-8], only *Empidomermis cozii* and *Reesimermis* (*Octomyomermis*) *muspratti*, have been described from mosquitoes in Africa [9-11]. *Octomyomermis muspratti* was isolated from various *Aedes* and *Culex* spp. larvae from tree holes in Livingstone, Zambia [11]. Subsequent colonies were established and *O. muspratti* was found to be capable of parasitizing *Aedes aegypti, Aedes polynesiensis, Culex pipiens, Anopheles stephensi*, and *Anopheles albimanus*[12]. *Empidomermis cozii* was found in adult *Anopheles funestus* from Burkina Faso [9,10].

Regardless of genera, aquatic mermithids that infect mosquitoes have similar life cycles. Eggs hatch in either standing or flowing water and the pre-parasitic forms attach to and bore into mosquito larvae. Parasitism is typically most successful when the pre-parasitic form invades a first or second instar mosquito. Pre-parasitic mermithids develop in the mosquito host, molt once, and emerge from the mosquito as post-parasitic forms. Mermithid emergence is typically lethal for the mosquito and may happen in the mosquito larval or adult stage. In the aquatic habitat the post-parasitic mermithids molt into adults, mate and lay eggs. The development time of each mermithid life stage varies among species, and this dictates at which mosquito life stage the post-parasitic form emerges [13].

Mosquito death upon mermithid emergence has sparked interest in using mermithids as biological control agents. From a classical biological control standpoint, Juliano (2007) cited several characteristics of mermithids that make them potentially successful biological control agents of mosquitoes: they are specialists, they develop in synchrony with mosquitoes, their populations can increase rapidly or be augmented by human re-inundation, and they require only one victim/host to complete their life cycle [14]. Platzer (2007) summarizes multiple characteristics of mermithids that make them attractive for biological control of mosquitoes, including ease of application, environmental safety, host specificity, laboratory manipulation of life history, lethality, mass rearing *in vivo*, and potential for long-term recycling in the environment. Numerous investigators have developed mass rearing techniques for mermithids [15-17] that can support large scale field releases. Mermithids have been applied to aquatic habitats as either pre-parasitic forms with a hand operated spray pump [17] or aerial spray pump [18] and post-parasitic forms applied to moist soil prior to flooding [19,20].

Major field releases of both *Romanomermis culicivorax* and *Romanomermis iyengari* have occurred for *Anopheles* population control. *Romanomermis iyengari* has been used to suppress malaria vector populations of *An. albimanus* in Cuba [21], *Anopheles pseudopunctipennis* in Oaxaca State, Mexico [22], and *Anopheles albitarsis* and *Anopheles rondoni* in Roraima State, Brazil [23]. *Romanomermis culicivorax* has been used to suppress malaria vector populations of *Anopheles freeborni* in California, USA [18,19], *An. albimanus* and *An. pseudopunctipennis* in El Salvador [16,24], *An. albimanus* in Mella Isla de la Juventud, Cuba [25], and *An. albimanus* in El Valle, Colombia [26]. The publication by Rojas *et al.* (1987) is notable because release of *R. culicivorax* may have both suppressed the vector population and reduced malaria prevalence in local children for up to two years post mermithid release. The successful control of *An. albimanus* by *R. culicivorax*, a mermithid that exits the mosquito in the larval stage, was facilitated by the fact that *An. albimanus* larvae occur in large standing bodies of water that can be readily identified and treated with mermithids [26]. This evidence demonstrates that mermithid nematodes can be successfully used as biological control agents of mosquitoes.

This report documents numerous mermithid nematodes that were observed in adult female *Anopheles* spp. collected during a malaria study conducted in southeastern Senegal [27]. Sequencing and phylogeny analysis were used to place the nematodes in the Mermithidae family and compare their relatedness to other mermithid species.

## Methods

**Figure 1:**
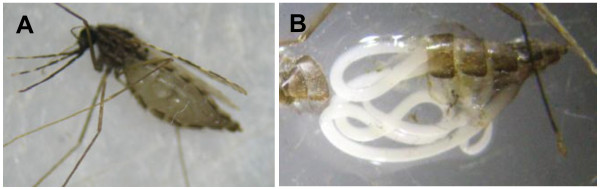
**Photograph of a mermithid-parasitized *An. rufipes * (A) and the dissected mosquito abdomen with mermithid parasite (B).**

All mosquitoes were collected in 2009 from the villages of Ndebou, Boundoucondi, Nathia, and Damboucoye in southeastern Senegal [28]. Blood fed mosquitoes were collected from huts during the mornings by backpack aspiration and held in a field insectary for up to five days [27]. Other mosquitoes were collected from CDC light traps hung next to bed nets; these mosquitoes were processed on the same day of capture. Mosquitoes were identified morphologically [29] and molecularly [30,31] to species. During routine morphological examination, mosquitoes were also inspected visually for possible mermithid infection. Adult mermithids range in length from 10 to 100 mm [1] so they can be readily observed without dissection of the mosquito (see Figure 1a). Mermithid nematodes were only observed in mosquito abdomens. Abdomens were separated from the thorax of the adult mosquitoes and stored on silica gel desiccant.

**Figure 2:**
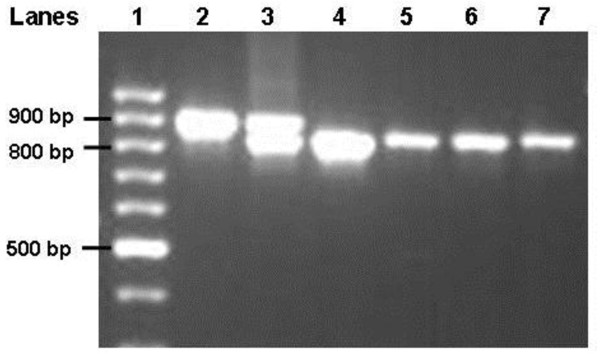
**Gel electrophoresis image of multiple samples amplified with mermithid primers: Lane 1 – 100 base pair ladder, Lane 2 – uninfected laboratory-reared  *An. gambiae * s.s., Lane 3 – Field-collected  *An. gambiae * s.s. infected with mermithid parasite, Lanes 4 – 7 – dissected mermithid samples from field-collected mosquitoes (free of *Anopheles * spp. tissue).**

Two mermithid-positive *An. gambiae* s.s. were dissected and the nematodes were separated from the mosquitoes so that mermithid DNA could be extracted with the Qiagen DNeasy kit (Qiagen Sciences, Maryland, USA) and a Qiacube robot (Qiagen Sciences, Maryland, USA). Primers were designed to amplify regions of the 18 S ribosomal RNA gene by the polymerase chain reaction (PCR). Primers were designed by alignment of sequences from other mermithids obtained from GenBank and making consensus primers. Primer sequences were: Merm forward – 5’-CAAGGACGAAAGTTAGAGGTTC-3’ and Merm reverse – 5’-GGAAACCTTGTTACGACTTTTA-3’. Twenty μl of a PCR enzyme mixture was used to amplify DNA from each sample; this mixture consisted of: 10 μl Fermentas 2X Master Mix, 2 μl forward and reverse primers at 10 μM, 2 μl DNA, and 4 μl of RNAse-free water. The thermocycler protocol was 5 minutes at 95°C, 30 seconds denaturing at 95°C, 30 seconds annealing at 50°C, and 45 seconds extension at 72°C for 40 cycles with an additional 10 minute extension step, all performed on a PTC-200 Peltier Thermal Cycler (MJ Research, Waltham, MA, USA). This protocol was used to assess the presence of mermithid DNA from the abdomens of blood fed mosquitoes that were processed for blood meal analysis [27]. The primers amplified an 804 base pair fragment of mermithid DNA and also a 900 base pair fragment of *Anopheles* spp. DNA. Thus, when these primers were used to screen wild *Anopheles* mosquitoes one band would always be present when a mosquito was processed, but when the mosquito was infected with a mermithid parasite, there would be two bands present (see Figure 2).

18 S rDNA amplicons from mermithid-only isolates were cloned in a TOPO-TA plasmid (Invitrogen, Carlsbad, CA, USA), plasmid DNA was harvested from transformed *E. coli* with a QIAprep Spin Miniprep Kit (Qiagen, Gaithersburg, Maryland, USA) and DNA was submitted for sequencing by the Proteomics and Metabolomics Facility at Colorado State University, Fort Collins, Colorado. Sequenced mermithid DNA was then used to identify 147 of the most similar sequences published in GenBank, based on E-score. Sequences were provisionally aligned using Clustal W [32]. This alignment was further adjusted by hand, based on the current understanding of RNA secondary models [33,34]. Maximum parsimony (MP) phylogenetic analysis was performed using the Phylogenetic Analysis Using Parsimony (PAUP) 4.0b10 package [35]. MP was chosen because it is the only phylogenetic method that uses gaps as phylogenetically informative characters in sequence alignments and gaps were common in the 18 S ribosomal RNA alignments. Four levels of outgrouping with 100 rounds of bootstrapping were performed. Nematodes from the Mermithidae family formed a monophyletic clade and therefore only sequences from these were compared for the final tree [GenBank Accession Nos.: *Agamermis changshaensis* – DQ628908.1, *Agamermis* sp. BH-2006 - DQ665653.1, *Agamermis xianyangensis* – EF617352.1, *Allomermis solenopsii* – DQ533953.1, *Amphimermis* sp. A-2007 – EF617354.1, *Amphimermis* sp. B-2007 – EF617355.1, *Cryptonchus tristis* – EF207244.1, *Dintheria tenuissima* – AY593942.1, *Gastromermis* sp. – AY146543.1, *Gastromermis* sp. BH-2006 – DQ533954.1, *Heleidomermis* sp. BH-2006 – DQ533955.1, *Hexamermis agrotis* – DQ530350.1, *Isomermis lairdi* – FN400900.1, *Mermis nigrescens* – AF036641.1, Mermithid sp. Jh-2004 – AY284743.1, Mermithidae sp. A-AV-2003 – AY374415.1, Mermithidae sp. C-AV-2003 – AY374417.1, Mermithidae sp. MHMH-2008 – FJ040480.1, *Octomyomermis huazangensis* – EF617353.1, *Ovomermis sinensis* – DQ520879.1, *Romanomermis culicivorax* – DQ418791.1, *Romanomermis sichuanensis* – EF612769.1, *Romanomermis wuchangensis* – DQ520878.1, *Strelkovimermis spiculatus* – DQ665654.1, *Thalassoalaimus pirum* – FJ040500.1, and *Thaumamermis cosgrovei* – DQ665655.1].

## Results

Mermithid DNA was amplified in a total of ten of 551 (1.8%) *Anopheles* spp. abdomens collected from southeastern Senegal. Mermithids were visually observed in four of these captured *Anopheles* spp. mosquitoes, and six others were detected only by PCR. Two of the four mermithid nematodes observed visually had emerged from the anus of aspirated mosquitoes that died during the five day holding period, similar to the description and photograph of *E. cozii* in Poinar (1977). One *An. rufipes* that had survived to five days post capture contained a live mermithid (Figure 1), but attempts to colonize the mermithid failed. Mermithid-positive *Anopheles* spp. were found from 3 of the 4 villages from which we sampled mosquitoes: Ndebou (5), Boundoucondi (3), and Damboucoye (2). Five of 74 (6.8%) *An. gambiae* s.s. tested from Ndebou, two of 91 (2.2%) *An. funestus* and one *An. rufipes* tested from Boundoucondi, and two of 51 (3.9%) *An. funestus* tested from Damboucoye were positive for mermithid parasites or mermithid DNA.

**Figure 3:**
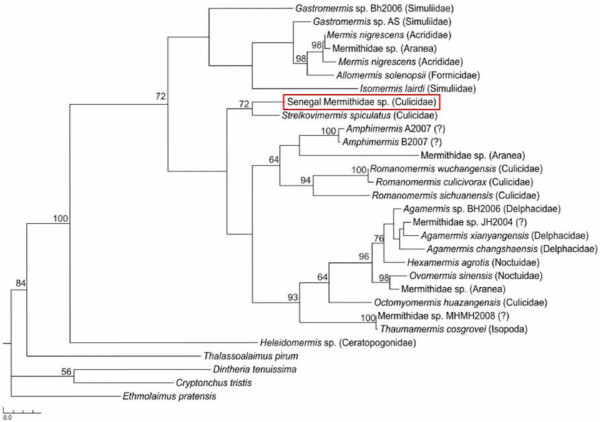
**The Maximum Parsimony tree demonstrates that the nematode found in various *Anopheles * spp. from Senegal is indeed a mermithid parasite.** Numbers at nodes represent the degree of bootstrap support. The scale represents the number of steps. Mermithid species names in italics are followed by the family or order the parasite was isolated from in parentheses.

Maximum parsimony analysis confirmed that the nematode parasites found in *Anopheles* from Senegal were indeed mermithid parasites. A clade containing Senegalese mermithids and *Strelkovimermis spiculatus* had 72% bootstrap support (Figure 3).

## Discussion

To our knowledge, this is the first report of a mermithid nematode from *Anopheles* mosquitoes in Senegal. The MP bootstrap analysis placed the Senegalese mermithid in the same clade with *Strelkovimermis spiculatus *. There are no *Empidomermis CulicimermisHydromermis*, or *Perutilimermis * 18 S rDNA entries in GenBank, therefore the exact placement of the Senegalese mermithid within the family Mermithidae is unknown. The two previous mermithid species isolated from Africa, *E. cozii *[9,10] and *O. muspratti*[11,12] do not have published 18 S ribosomal RNA sequences. The distance of the Senegalese mermithid from *Octomyomermis huazangensis* in the MP tree (Figure 3) suggests that this parasite does not belong to the *Octomyomermis * genera. It is possible that the Senegalese mermithids were re-isolations of *E. cozii * or a different mermithid species not listed in GenBank and therefore not a novel mermithid parasite isolation. The Merm primers developed here could be useful for screening mosquito populations for novel mermithid isolates. Re-isolation of the Senegalese mermithid and proper preparation of specimens for taxonomic evaluation will be necessary to make a more accurate species identification.

Only two *Strelkovimermis * species have been identified as parasites of mosquitoes, *Strelkovimermis spiculatus *[8] and *Strelkovimermis peterseni*[36]. Both *S. spiculatus*[37] and *S. peterseni*[36] have only been reported to exit the mosquito in the larval stage. *Strelkovimermis spiculatus* parasitize *Anopheles* species in laboratory settings but not as efficiently as *Culex* and *Aedes* species [37,38]. Conversely, *S. peterseni* was only capable of parasitizing *Anopheles* species larvae and not any of the *CulexAedes*, or *Psorophora* larvae tested [39]. Similar to other mermithid species, non-target aquatic arthropods were rarely parasitized by *S. spiculatus*, and therefore *Strelkovimermis* species may be desirable for use as a biological control agent [37,38]. To date, no field release of either *S. spiculatus* or *S. peterseni* has occurred [13].

The Senegalese mermithid may have life cycle characteristics similar to *Empidomermis* spp. Three isolations of *Empidomermis* spp. have been documented: *E. cozii* from Burkina Faso [9,10], *Empidomermis riouxi* from France [40], and *Empidomermis* spp. from New York, USA [41]. *Empidomermis cozii* was reported to emerge from the anus of adult mosquitoes [10], similar to what we observed with the Senegalese mermithid. *Empidomermis cozii* cannot fully develop in the adult mosquito unless a blood meal has been ingested by the mosquito [10], and all of the mermithid-positive mosquitoes during our study were blood-fed at the point of capture.

Senegalese mermithids were observed or detected in roughly 2% of the *Anopheles* in our study area which demonstrates that mermithids are naturally present in *Anopheles* populations in West Africa. The Merm primers amplified both mosquito and nematode DNA, therefore sequence data was only available for mermithids that had emerged from the adult mosquito and it is not possible to tell if all mermithids detected were the same species. Mermithid parasites were found in *An. gambiae* s.s., *An. funestus*, and *An. rufipes.* If these mermithid specimens were the same nematode species in all three mosquito species, this would indicate that the mermithid has a broad host range within the *Anopheles* genera. In the future, additional sequencing will be necessary to determine if the Senegalese mermithids from all infected *Anopheles* species are the same mermithid species or possibly distinct molecular variants as was observed with *Mesomermis flumenalis* in multiple *Simulium* species [42]. *Anopheles gambiae* s.s., *An. funestus*, and *An. rufipes* inhabit a diverse and disparate range of larval habitats with typically little overlap [43], therefore the transmission of the Senegalese mermithid may not be restricted by larval habitat type. A mermithid with a broad mosquito host range is more likely to be recycled in the environment which would reduce the number of releases necessary for biological control of mosquito populations [13].

Several characteristics of the development of *Empidomermis* and the Senegalese mermithid described here may make it an ideal biological control agent for the African malaria vector *Anopheles gambiae*. Larval control of *Anopheles gambiae* is difficult because its larval habitats are extremely abundant, ephemeral or inaccessible [44]. A mermithid parasite that emerges from the adult could be dispersed to cryptic *An. gambiae* larval habitats. Biological control of mosquito larvae in ephemeral larval habitats is difficult due to issues of larval density dependence, compensatory mortality, and overcompensation [14]; however, a mermithid that emerges from the adult mosquito would bypass these issues of biological control. Furthermore, mermithid parasites that emerge from the adult mosquito may be more effective for vector-borne disease control than a mermithid that emerges from a larval mosquito as this is likely to reduce the daily probability of adult survival, the most critical variable in the vectorial capacity equation [45]. It was speculated that *Isomermis lairdi*, a mermithid parasite of *Simulium damnosum* that emerges in the adult life stage of the black fly, may limit *Onchocerca volvulus* transmission by killing the adult black fly vector before *O. volvulus* could fully develop and be transmitted [46]. All three *Empidomermis* species were reported to sterilize adult mosquitoes [9,40,41]. *Empidomermis*-parasitized females had arrested ovarian development [40,41] and inhibited mating activity of parasitized males [40]. These characteristics would maximize transmission of the mermithid to other mosquito offspring as opposed to infection of the parasitized females own offspring. For ideal mermithid-based control of malaria parasite transmission, the mermithid would need to emerge from the adult mosquito prior to *Plasmodium* completion of the extrinsic incubation period in the *Anopheles* vector. There is also the possibility that the mermithid may inhibit *Plasmodium* development in the mosquito, which could further impact vector-borne disease control. While a mermithid that emerges from the mosquito adult stage may be more difficult to rear in large numbers for mass release, this same life history trait may be used to facilitate a novel, rapid dispersal method of the mermithid into the environment if mermithid-infected adult mosquitoes were released.

The re-isolation of the Senegalese mermithid from adult mosquitoes should be feasible. Specimens should be prepared and submitted to a nematode taxonomist to make a formal identification and description of the species. Laboratory colonies should be established that utilize rearing techniques published for several other mermithids from mosquitoes [15,17]. Sampling of wild adult and larval mosquito populations irrespective of mosquito genera over a longer duration of time combined with DNA barcoding of mermithids will determine critical information on host and temporal range of the mermithid. The life cycle and interactions of the Senegalese mermithid with various *Anopheles* species should be determined to assess the potential effect that mermithid parasitism may have on vector populations and malaria parasite transmission in order to define the utility of the mermithid as a biological control agent.

## Conclusions

To our knowledge, this is the first report of mermithids from adult *Anopheles* mosquitoes in Senegal. Mermithids were present in 1.8% of the sampled adult *Anopheles* species in our study area. The mermithid was found in *An. gambiae* s.s., *An. funestus*, and *An. rufipes* which suggests parasite infection occurred in numerous, diverse mosquito larval habitats. Although maximum parsimony analysis determined the mermithid was closely related to *S. spiculatus*, several characteristics of the Senegalese mermithid were more similar to those more commonly associated with the *Empidomermis* genus, such as emergence from the adult mosquito. Future mermithid isolations of the Senegalese mermithid will hopefully allow for formal taxonomic identification, laboratory colonization, determination of life history traits and species specificity, and characterize its usefulness as a biological control agent.

## Competing interests

The authors declare no competing interests.

## Authors’ contributions

KCK collected and processed specimens, analyzed data, and wrote the manuscript. MS collected specimens and wrote the manuscript. WBIV performed the phylogenetic analyses and wrote the manuscript. BDF collected specimens, provided reagents, and wrote the manuscript. All authors read and approved the final version of the manuscript.
